# Isoscopoletin inhibits hepatocellular carcinoma cell proliferation via regulating glycolysis-related proteins

**DOI:** 10.1371/journal.pone.0310530

**Published:** 2024-11-07

**Authors:** Chenyao Ruan, Chen Wang, Jiawen Gu, Zhihui Zhu

**Affiliations:** 1 School of Pharmaceutical Sciences, Zhejiang Chinese Medical University, Hangzhou, China; 2 College of Pharmacy, Hubei University of Chinese Medicine, Wuhan, China; University of Chicago, UNITED STATES OF AMERICA

## Abstract

**Objective:**

Isoscopoletin is one of the primary metabolites of natural product scoparone, which was reported to against tumor proliferation. The aim of this study was to explore the mechanism of isoscopoletin against hepatocellular carcinoma (HCC).

**Methods:**

Transcriptomics was used to reveal the possible pathways of isoscopoletin against HCC *in vitro*. The potential targets of isoscopoletin against HCC through affecting glycolysis were analyzed by network pharmacology, then the potential binding abilities of isoscopoletin to glycolysis-related proteins were initially verified by high throughput virtual molecular docking. The affinities of isoscopoletin for glycolysis-related proteins were assayed using microscale thermophoresis (MST), which was reverse-validated by inhibiting the binding ability of isoscopoletin to GPD2. Glucose consumption and lactate production were examined to evaluate the effects of isoscopoletin on intracellular glycolysis, and the regulation of glycolysis-related targets by isoscopoletin was detected using RT-qPCR and ELISA kits.

**Results:**

The results of transcriptomics showed that the differentially expressed genes (DEGs) were mainly enriched in glycolysis and other metabolic-related pathways. Network pharmacology and molecular docking revealed that GPD2, GPI, HSP90AA1 and PGK2 were the core targets in the glycolysis process of isoscopoletin against HCC. MST results showed that there was a strong affinity between isoscopoletin and GPD2, GPI, Hsp90α and PGK2. *In vitro* results showed that isoscopoletin inhibited glucose consumption and lactate production, while regulating the levels of glycolysis-related proteins.

**Conclusion:**

This study suggests that isoscopoletin may exist an anti-tumor effect by regulating the glycolysis-related proteins GPD2, GPI, Hsp90α and PGK2, inhibiting the glycolysis process in HCC cells, then blocking the energy supply of tumor cells.

## Introduction

Along with the development of tumor metabolism study, abnormal tumor metabolism has already been identified as an important marker for tumors. The metabolic shift from oxidative phosphorylation to the Warburg effect satisfies the need for tumor cells to proliferate rapidly and provides a beneficial microenvironment for tumor development [1]. Understanding the mechanism of Warburg effect regulation in HCC can enrich our insights into HCC and lay the foundation for the search of novel diagnostic biomarkers and clinical therapeutic targets for HCC.

According to the Warburg effect: compared to normal cells, cancer cells produce large amounts of ATP (up to 60%) by glycolysis for nutrient supply even when they are hypoxic [2]. Aerobic glycolysis metabolizes glucose hundred times faster than the complete oxidation of glucose in mitochondria [3]. Glycolysis, as a well-established metabolic pathway, provides a favorable microenvironment for tumor cell growth and metastasis, in addition to providing ATP for cancer cell metabolism. Aerobic glycolysis has been used as a hallmark of HCC [4], therefore, inhibition of glycolysis energy in cancer has been considered as one of the anti-cancer strategies. Glycolysis is accomplished through a series of enzymatic reactions in the cytoplasm, and the increased expression and activity of its associated enzymes are essential in tumor cells, among which GPD2, GPI, Hsp90α and PGK2 are involved in promoting glycolysis and cancer cell proliferation [5, 6]. These enzymes may be the potential biomarkers and targets for tumor diagnosis and therapy.

Scopoletin and isoscopoletin are the major primary metabolites of scoparone. Scoparone is a coumarin, which has been reported as a therapeutic drug in liver diseases [7], such as acute liver injury [8, 9], non-alcoholic fatty liver disease [10, 11], fibrosis [12], it has been reported that scoparone has a good inhibitory effect on pancreatic cancer [13], prostate cancer [14] and melanoma [15]. And scopoletin has been reported to be used for against Non-small cell lung cancer [16], HCC [17], human cervical cancer [18], breast cancer [19] and other cancers [20]. As another metabolite, isoscopoletin has been reported to have an IC_50_ value of 4.0 μM in human CCRF-CEM leukaemia cells without drug resistance, which indicates its excellent anti-tumor activity [21]. In addition, isoscopoletin has shown favorable inhibitory activity against lung cancer and colon cancer *in vitro* [22], however its anti-cancer mechanism is not clear.

MST is the latest hypersensitive technique for analyzing intermolecular interactions. It realizes the quantitative measurement of intermolecular affinity by monitoring the directional movement of molecules in the microscopic temperature gradient. MST uses fluorescence as a means of monitoring and analysis. It is sensitive and rapid, does not need molecular surface immobilization, only needs μL level of sample size, and has no restriction on the relative molecular weight and molecular size of the tested substance. It can also be determined in any buffer or even in cell lysate or serum, and the detection sensitivity can be as low as pM level [23–26]. MST is an important and powerful tool used in biomedical field, such as disease diagnosis, medical laboratory and basic medical research, drug development and signaling pathway research.

In this study, based on the results of transcriptomics, the potential targets and pathways of isoscopoletin against HCC were screened by network pharmacology, the binding activity of isoscopoletin to potential targets was detected by virtual molecular docking and MST, then the effect and mechanism of isoscopoletin in inhibiting glycolysis against HCC were verified *in vitro*, which provided a novel direction for the possible targets and mechanism of isoscopoletin against HCC.

## Materials and methods

### Chemical and reagents

Isoscopoletin (PubChem CID: 69894) was provided by Beijing Century Aoke Biology Research Co., Ltd, China (concentration ≥ 98%). TRIzol™ reagent (15596018CN) and DyLight^®^488 fluorescent dye (L32470) were purchased from Thermo Fisher Scientific (Massachusetts, USA). Recombinant DNase I (2270A) was purchased from Takara Biomedical Technology Co., Ltd. (Beijing, China). HiFi-MMLV cDNA Kit (CW0744) was purchased from Cowin Bio. (Beijing, China). Microscale Thermophoresis (NT. 115) was from Nano Temper (Munich, Germany). GPD2 (ab132692), GPI (ab116799), Hsp90α (ab78425), PGK2 (ab123166) and recombinant anti-GPD2 antibody (ab188585) were purchased from Abcam (Cambridge, UK). Glucose kit (A006-1-1) and Lactate kit (A019-2-1) were purchased from Jiancheng Institute of Biological Engineering (Nanjing, China). GPD2 ELISA kit (EF009945) was purchased from WeiKeSaiSi Technology Co., Ltd. (Wuhan, China). GPI ELISA kit (CSB-E09589h) and Human Heat shock protein HSP 90-beta ELISA kit (CSB-EL010808HU) were purchased from Huamei Biological Engineering Co., Ltd. (Wuhan, China). PGK2 ELISA kit (CB15537393) was purchased from Shanghai Yanmu Industrial Co., Ltd. (Shanghai, China).

### Cell culture

The human hepatoma cell line HepG2 (purchased from Cell Bank of the Chinese Academy of Sciences, Shanghai, China) was inoculated into 6-well plate according to 5 × 10^5^ cells/well. After the cells adhered to the wall, the culture medium was discarded, 2 mL fresh medium was replaced for the control group, and 2 mL medium containing 10 μM isoscopoletin extract was replaced for the drug treatment group. Then the 6-well plate was cultured at 37°C and 5% CO_2_ for 24 h.

### Total RNA extraction

The culture medium in the 6-well plate was discarded and the underlying cells were collected. The total RNA of each group was extracted from with or without TRIzol™ reagent according to the RNA extraction instructions, and the genomic DNA was removed by DNase Ⅰ. The RNA quality was measured using 2100 bioanalyzer (Agilent Technologies, Inc., California, USA), then the RNA quality analysis was carried out with Nano Drop-2000 (Thermo Fisher Scientific, Massachusetts, USA) to ensure that OD_260/280_ = 1.8–2.2, OD_260/230_ ≥ 2.0, which was used to construct the sequence library.

### Transcriptome sequencing

Using TruSeq™ RNA sample preparation equipment (Illumina, California, USA) to separate mRNA from 5 μg total RNA. Using oligo (dT) bead to separate and fragment the messenger RNA according to the ployA selection method. Double-stranded cDNA was synthesized by HiFi-MMLV cDNA Kit and random hexamer primers. According to the library construction scheme, the synthesized cDNA was repaired, phosphorylated and ’A’ base added. The target cDNA fragment of 200-300bp was selected for 15 cycles of PCR amplification. The paired RNA-seq sequencing library was sequenced by Illumina HiSeq xten (2 × 150 bp).

The filtering of the original data mainly includes removing reads with adapter, removing reads containing N (N means that the base information cannot be determined), and removing low-quality reads (reads with Qphred ≤ 20 bases accounting for more than 50% of the entire read length). Meanwhile, the contents of Q20, Q30 and GC in clean data were calculated. All subsequent analyses were based on high-quality clean data.

### Differential expression analysis

The expression of genes was quantitatively analyzed to determine the differential expression of genes between different samples, combined with the information of sequence functional annotation to reveal the regulatory mechanism of genes. After the gene count was obtained, the differential expression analysis of gene was performed on the control group and experimental group to identify the DEGs between samples [27].

In order to identify the DEGs between two different samples, the expression level of each transcript was calculated by the method of FRKM [28]. The abundance of gene was quantified by RSEM v1.3.3 (http://deweylab.github.io/RSEM/). Then, the R software statistical package EdgeR (Empirical analysis of digital gene expression data in R, https://www.bioconductor.org/packages/2.12/bioc/html/edgeR.html) was used to analyze the differential expression. Using Fisher accurate test, the *P* value was verified by multiple calibration methods such as BH (Benjamini and Hochberg) and BY (Benjamini and Yekutieli), and the modified *P* value was set < 0.05. The correlation was sorted according to the *P* value. The greater the log_2_FC, the greater the difference in the expression of up-regulated genes, the smaller the log_2_FC, the greater the difference in the expression of down-regulated genes; the closer log_2_FC to 0, the smaller the differential expression ratio of genes.

### Prediction of intersection targets

Based on the results of transcriptomics, PharmMapper (http://www.lilab-ecust.cn/pharmmapper/) and SwissTargetPrediction (http://www.swisstargetprediction.ch/) were used to predict the targets of isoscopoletin. Using MalaCrads (https://www.malacards.org/) and GeneCards (https://www.genecards.org/) to obtain the currently reported disease targets extremely related to HCC. The intersection targets of isoscopoletin and HCC could be considered as the potential targets of isoscopoletin against HCC.

### Intersection targets enrichment analysis

The intersection targets were imported into DAVID database (https://david.ncifcrf.gov/) for Gene Ontology (GO) function and Kyoto Encyclopedia of Genes and Genomes (KEGG) signaling pathway enrichment analysis. The Annotation results of KEGG signaling pathways suggested that pathways with more intersection targets and smaller *P*-value could be considered as important pathways for the regulation of isoscopoletin against HCC [29].

### Protein-protein interaction and "Isoscopoletin-Target-Pathway" network

The intersection targets were uploaded to the STRING database (https://cn.string-db.org/) to construct the protein–protein interaction (PPI) network. The STRING database includes numerous known or predicted PPI relationships [30]. In addition, proteins suitable for target analysis were obtained by comparing the literatures. Cytoscape 3.9.1 software (https://cytoscape.org/) was used to construct the "Isoscopoletin-Target-Pathway" network. Intersection targets and important pathways were inputted as nodes and if a connection existed between two nodes, an edge was added to show the connection [31].

### Molecular docking

In order to study the interaction between isoscopoletin with potential targets, virtual docking was performed by Discovery Studio 2019 Client software (http://www.discoverystudio.net/). The 3D ligand file of isoscopoletin was obtained from PubChem (https://pubchem.ncbi.nlm.nih.gov/). The 3D structure of the potential proteins were downloaded from the Protein Data Bank (PDB, https://www.rcsb.org/).

Imported the protein structure into Discovery Studio 2019 Client software, Hetatm and Ligand groups were removed, and then cleaned the protein. After all receptor cavities were exposed and the radius of each site was adjusted to surround isoscopoletin, then the protein could be docked with isoscopoletin. It is generally believed that the larger the LibDock Score, the stronger the binding effect of isoscopoletin with proteins. And only when the LibDock Score ≥ 90.0 between isoscopoletin and proteins, it is considered to have a docking effect [32].

### The binding of isoscopoletin to glycolysis-related proteins

To determine the binding and magnitude of affinity of isoscopoletin with the glycolysis-related proteins screened by molecular docking, each 100–500 μL protein solution was labeled with 50 μg fluorescent dye DyLight^®^488 according to the requirements of the MST assay. And different assay conditions, including LED intensity and test temperature, were set for different samples. In the process of fluorescent labeling, proteins might be affected, and SD-test was used to detect the effect of fluorescence on proteins.

The change of fluorescence signal during the thermophoretic motion in the temperature gradient field after the affinity between isoscopoletin and glycolysis-related proteins was detected, and the influence of antibody on interaction between isoscopoletin and GPD2 was investigated by adding antibody to the solution in the MST experiment. All the measured values were multiplied by 1000 and converted into the relative change of fluorescence per thousand times, and the dissociation constant was calculated by the normalized fluorescence difference between the bound and unbound states, so as to analyze the affinity between isoscopoletin and the cancer-related proteins. The fluorescence intensity measured by MST was used to fit the value, and *K*_d_ value was calculated.

### Anti-HCC effect of isoscopoletin *in vitro*

After incubated with 10 and 40 μM isoscopoletin, the relative cellular viability of HepG2 cells was determined by MTT assay, and intracellular glucose consumption and lactate production were detected according to the instructions of the biochemical kit. The mRNA levels of glycolysis-related genes were measured by RT-qPCR, while the protein expressions were measured by ELISA kits.

Total RNA from HepG2 cells and cushed liver tissue was extracted using Trizol, and 1.0 μg of RNA was reverse transcribed using a cDNA synthesis kit according to the manufacturer’s instructions. The quantitative real-time polymerase chain reaction was carried out using SYBR Green PCR Master Mix (Bio-rad, USA).

The primers used for RT-qPCR were as follows: GDP2-F: 5’-ATCTTGCTGCCACCTATG-3’; GDP2-R: 5’-CACCTCTGCTTCAATGTATG-3’. GPI-F: 5’-CCGCGTCTGGTATGTCTCC-3’; GPI-R: 5’-CCTGGGTAGTAAAGGTCTTGGA-3’. HSP90AA1-F: 5’-CCCAGAGTGCTGAATACCCG-3’; HSP90AA1-R: 5’-TAACAGGTGCCCTGCTTCTC-3’. PGK2-F: 5’-AAGCCTTCCGAGCATCAC-3’; PGK2-R: 5’-CCACCAAGTATAGCCAGAAAG-3’. GAPDH-F: 5’-AGGTCGGTGTGAACGGATTTG-3’; GAPDH-R: 5’-GGGGTCGTTGATGGCAACA-3’. The relative target gene mRNA expression was determined by the 2^−ΔΔCt^ method and normalized against GAPDH mRNA levels.

### Statistical analysis

Data were analyzed by SPSS 16.0 software and expressed as mean ± SD. Comparison between groups was considered statistically significant when *P* < 0.05 by one-way ANOVA.

## Results

### Enrichment analysis of DEGs

**Fig 1:**
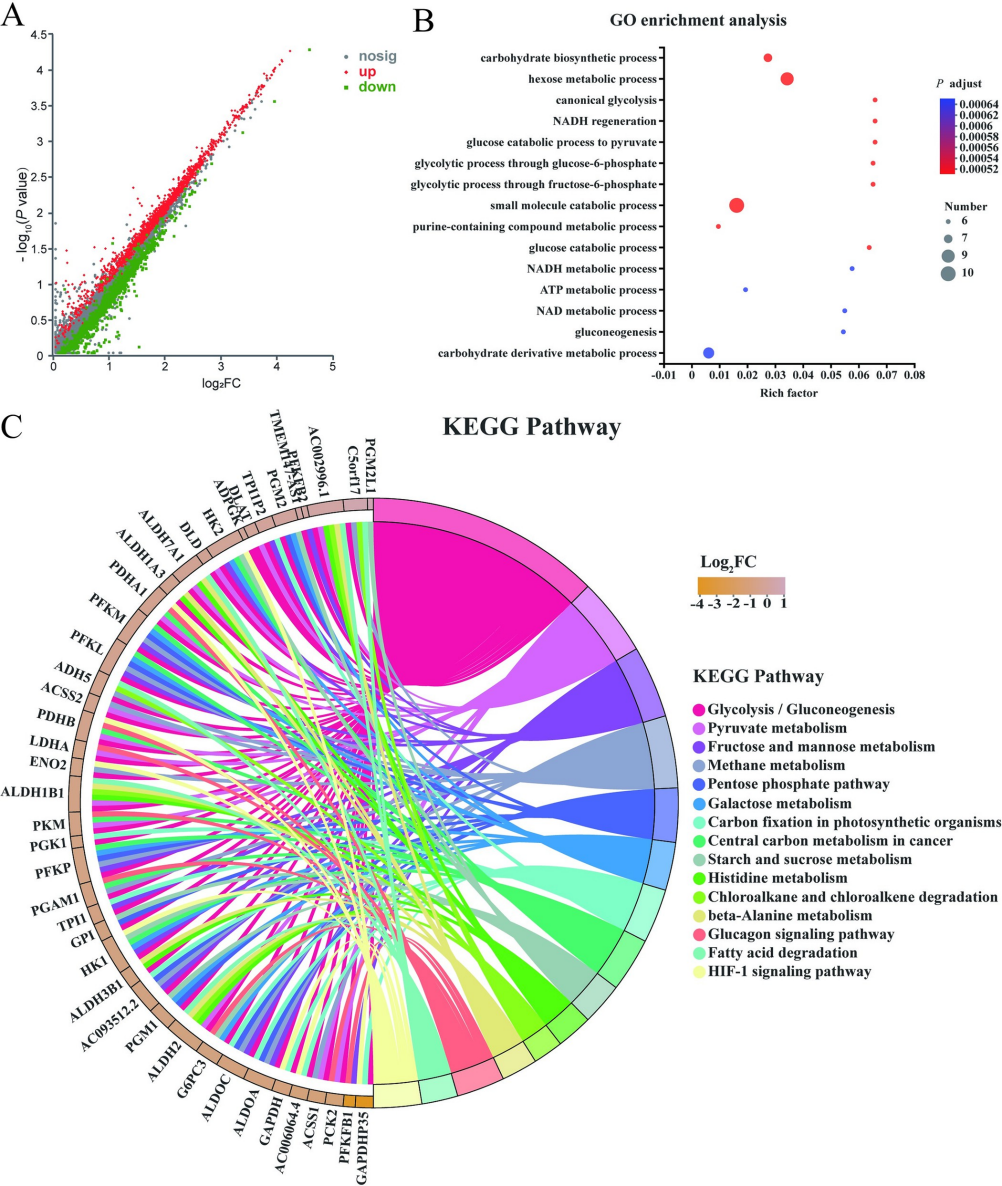
The results of transcriptome sequencing (n = 3). (A) The scatter diagram of DEGs. The red dots represent the up-regulated genes, the green dots represent the down-regulated genes, and the gray dots represent the non-significantly DEGs. (B) Bubble chart of GO enrichment analysis. (C) String diagram of KEGG pathway enrichment.

The scatter diagram of DEGs was shown in Fig 1A. GO function and KEGG signaling pathway enrichment analysis of DEGs were carried out by Goatools 1.4.4 software (https://pypi.org/project/goatools/).

Fig 1B showed that the DEGs were significantly enriched in carbohydrate biosynthetic process, hexose metabolic process and canonical glycolysis, respectively. According to the size of the dots, it was found that most of the DEGs were enriched in glycolysis-related biological process. According to the color of the dots, the *P* value of the first 15 function annotation results was small and reliable.

The pathways which met the conditions were selected for KEGG analysis, and the DEGs induced by isoscopoletin were enriched in different signaling pathways. The first 15 signaling pathways were shown in Fig 1C. The results showed that DEGs were significantly enriched in glycolysis/gluconeogenesis, pyruvate metabolism, fructose and mannose metabolism and other metabolism and energy pathways. The signaling pathway of DEGs enrichment focused on tumor-specific glycolysis-related molecular pathways, which may be the potential mechanism of the anti-tumor effect of isoscopoletin.

### Intersection targets prediction and analysis

Based on the results of transcriptomics, 243 isoscopoletin-related targets were obtained from PharmMapper and SwissTargetPrediction, and 385 HCC-glycolysis-related targets were screened out from MalaCards and GeneCards. And then the isoscopoletin target genes and HCC target genes were analyzed and compared by Veen online software. The information of the 50 intersection targets were listed in S1 Table. These intersection targets could be regarded as the potential targets in the treatment of isoscopoletin against HCC.

**Fig 2:**
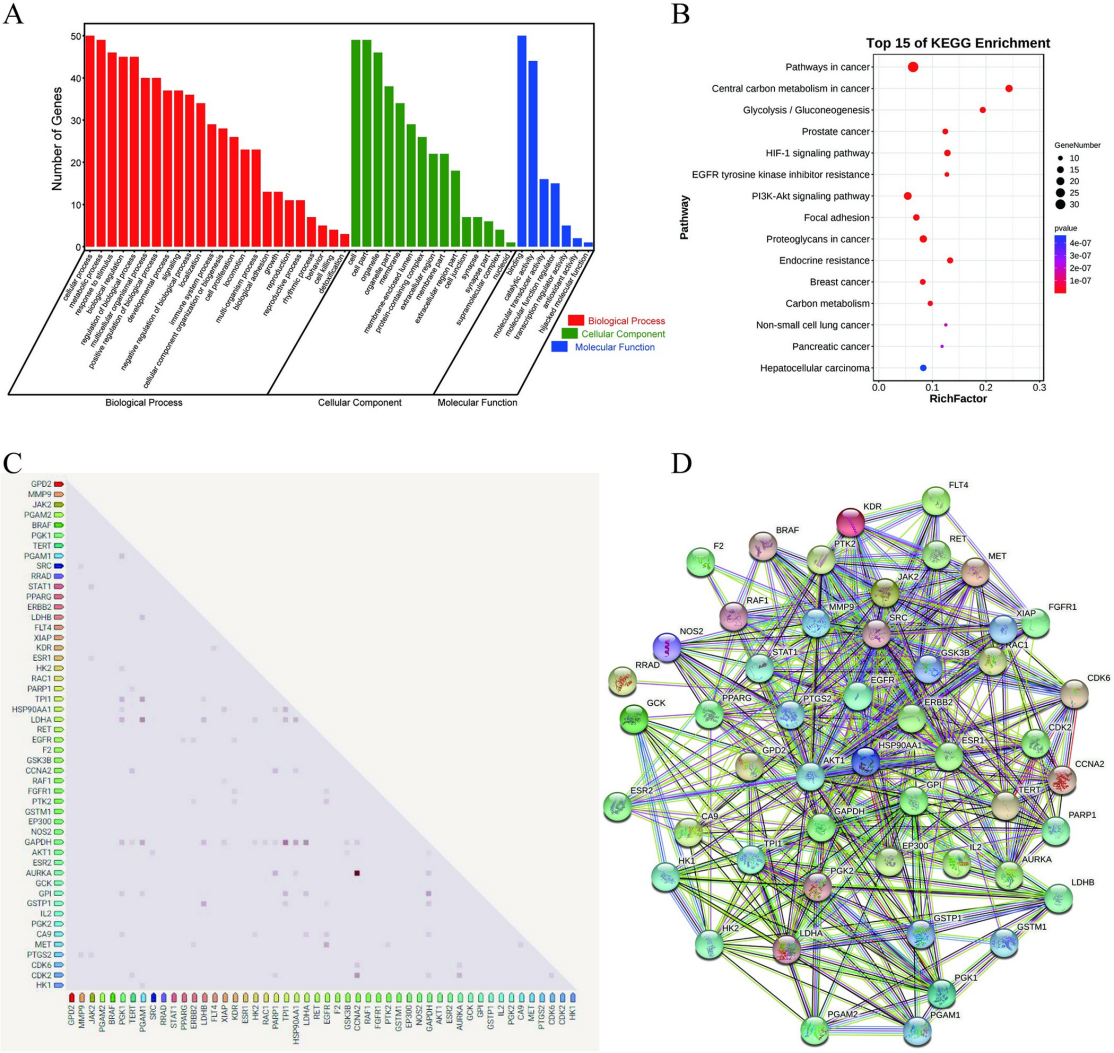
The results of intersection targets enrichment analysis and PPI networks. (A) GO enrichment analysis. (B) Bubble chart of KEGG pathway enrichment analysis. (C) Co-expression scores based on RNA expression patterns, and on protein co-regulation provided by ProteomeHD. (D) PPI network.

All the intersection targets were imported to DAVID database, and the threshold *P* ≤ 0.05 was set for GO function and KEGG pathway enrichment analysis. The results were shown in Fig 2A and 2B. Fig 2A showed that GO function enrichment mainly involved in biological process (BP), cellular component (CC) and molecular function (MF). BP enrichment focused on metabolic process, response to stimulus, biological regulation and other processes. CC enrichment showed that the proteins focused on organelle, membrane, synapse and other intracellular region. MF enrichment focused on binding, catalytic activity, molecular transducer activity and other functions. Fig 2B showed that these intersection targets mainly involved in pathways in cancer, central carbon metabolism in cancer, glycolysis/gluconeogenesis and other signaling pathways.

### PPI and "Isoscopoletin-Target-Pathway" network construction

All the intersection targets were imported into STRING database to construct the PPI network. Proteins which could interact with other proteins were ranked according to the Degree value. The higher the Degree value, the stronger the interaction of protein-protein, as shown in Fig 2C, 2D and Table 1. The results showed that GAPDH, AKT1, HSP90AA1, EGFR, ERBB2 and other proteins played a central role in the PPI network, and these proteins had the strongest interactions with other proteins.

**Table 1:** The degree value of PPI and "Isoscopoletin-Target-Pathway" network.

PPI		Network	
Gene	Degree	Gene	Degree
GAPDH	44	AKT1	14
AKT1	36	EGFR	14
HSP90AA1	36	ERBB2	13
EGFR	33	GPI	12
ERBB2	31	HSP90AA1	11
SRC	31	RAF1	11
ESR1	29	GPD2	10
MMP9	25	PGK2	10
PPARG	23	BRAF	9
PTGS2	22	MET	9

**Fig 3:**
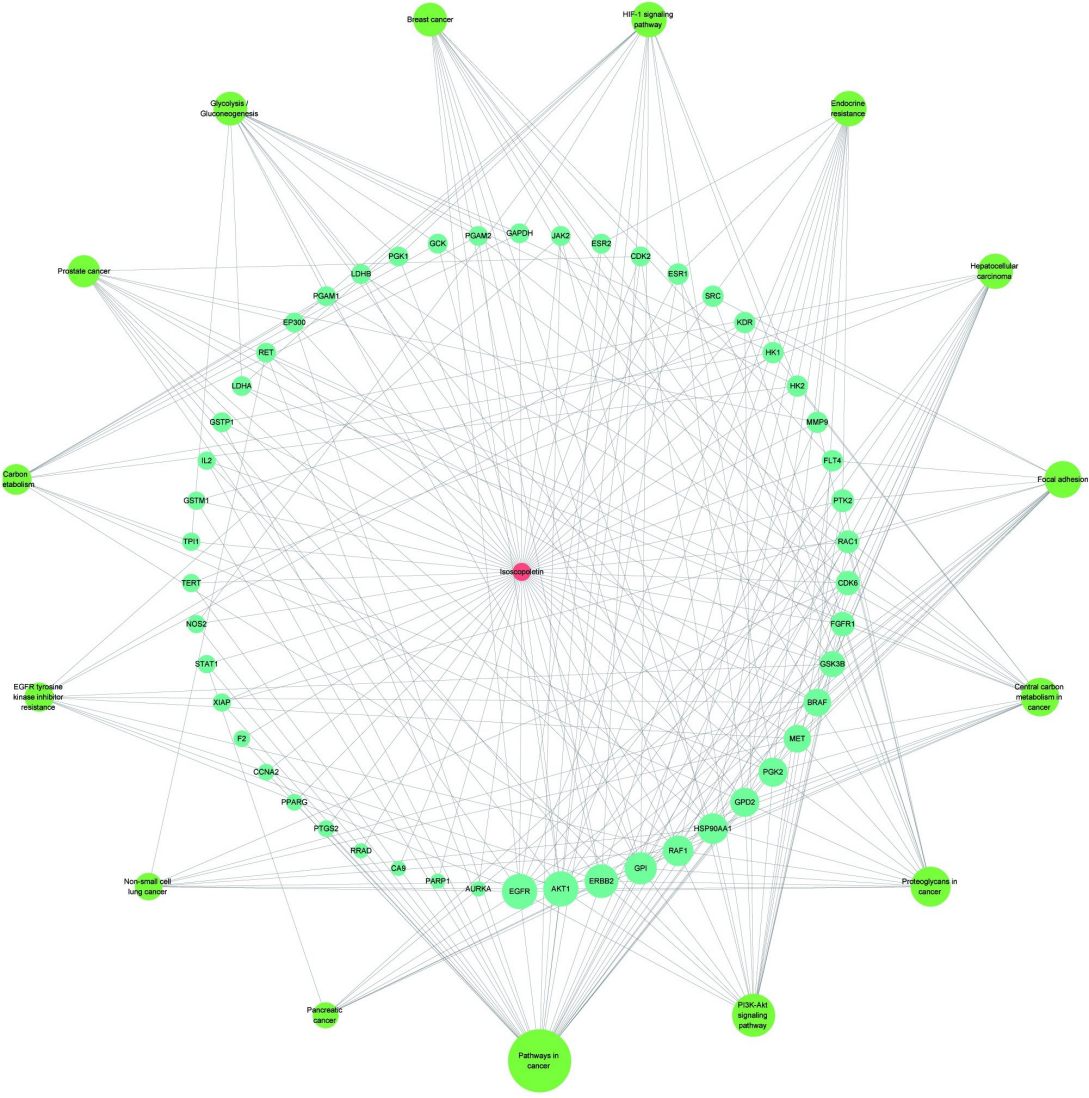
"Isoscopoletin-Target-Pathway" network.

50 intersection targets, the top 15 signaling pathways, as well as isoscopoletin were used to construct the "Isoscopoletin-Target-Pathway" network, as shown in Fig 3. In the network, red nodes represent isoscopoletin, blue nodes represent the intersection targets, and green nodes represent the selected pathways, the interaction between nodes was represent by line segment. Similarly, the Degree value was used to represent the number of line segments, that is, the number of connections between nodes, which was shown in Table 1. Obviously, AKT1, EGFR, ERBB2, GPI, HSP90AA1 and other targets had higher Degree value, suggesting that they might be the potential targets of isoscopoletin against HCC. Through the analysis of PPI and the "Isoscopoletin-Target-Pathway" network, 16 potential targets were screened out for molecular docking experiment.

### Molecular docking and analysis

**Fig 4:**
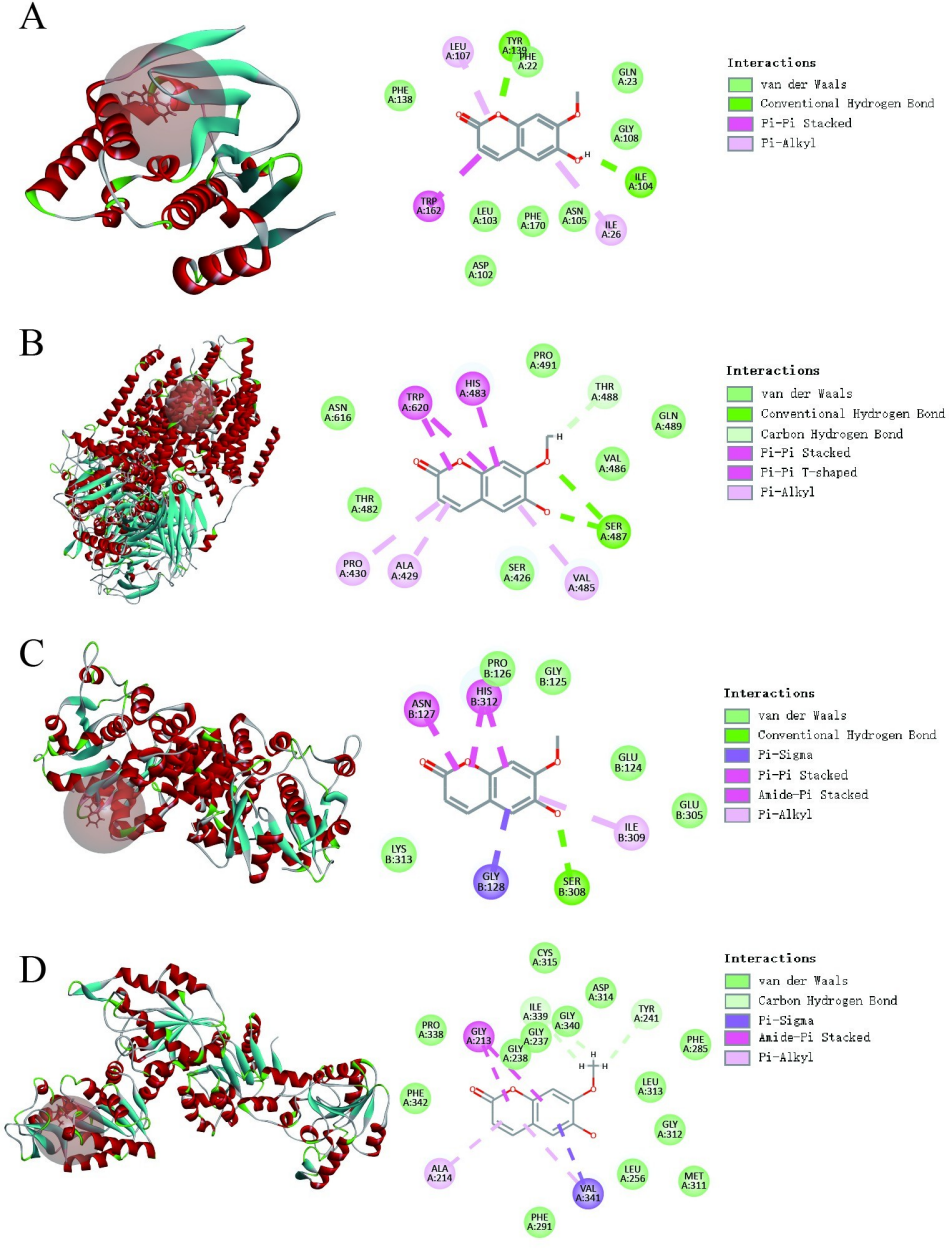
The results of molecular docking. (A) Isoscopoletin-Hsp90AA1 (5njx). (B) Isoscopoletin-GPI (7w72). (C) Isoscopoletin-GPD2 (1x0v). (D) Isoscopoletin-PGK2 (2paa).

Molecular docking software was used to conduct virtual docking study on isoscopoletin and the 16 potential targets screened above. The LibDock scores of isoscopoletin with these potential targets were shown in Fig 4 and Table 2. The results showed that the LibDock scores of isoscopoletin with GPD2, GPI, HSP90AA1 and PGK2 were all greater than 90, suggesting that isoscopoletin has a strong binding ability with GPD2, GPI, HSP90AA1 and PGK2, but the specific binding ability needs to be further verified.

**Table 2:** The LibDock score of isoscopoletin with potential targets.

Genes	LibDock Score
GPD2	98.0774
GPI	97.4651
HSP90AA1	92.6032
PGK2	90.3647
MMP9	88.6221
ERBB2	83.4271
PTGS2	81.4144
PPARG	80.9753
EGFR	80.5967
AKT1	79.1322
SRC	77.6930
RAF1	76.0744
ESR1	74.7604
GAPDH	71.9200
BRAF	66.9141
MET	Failed

### The binding of isoscopoletin to core proteins

**Fig 5:**
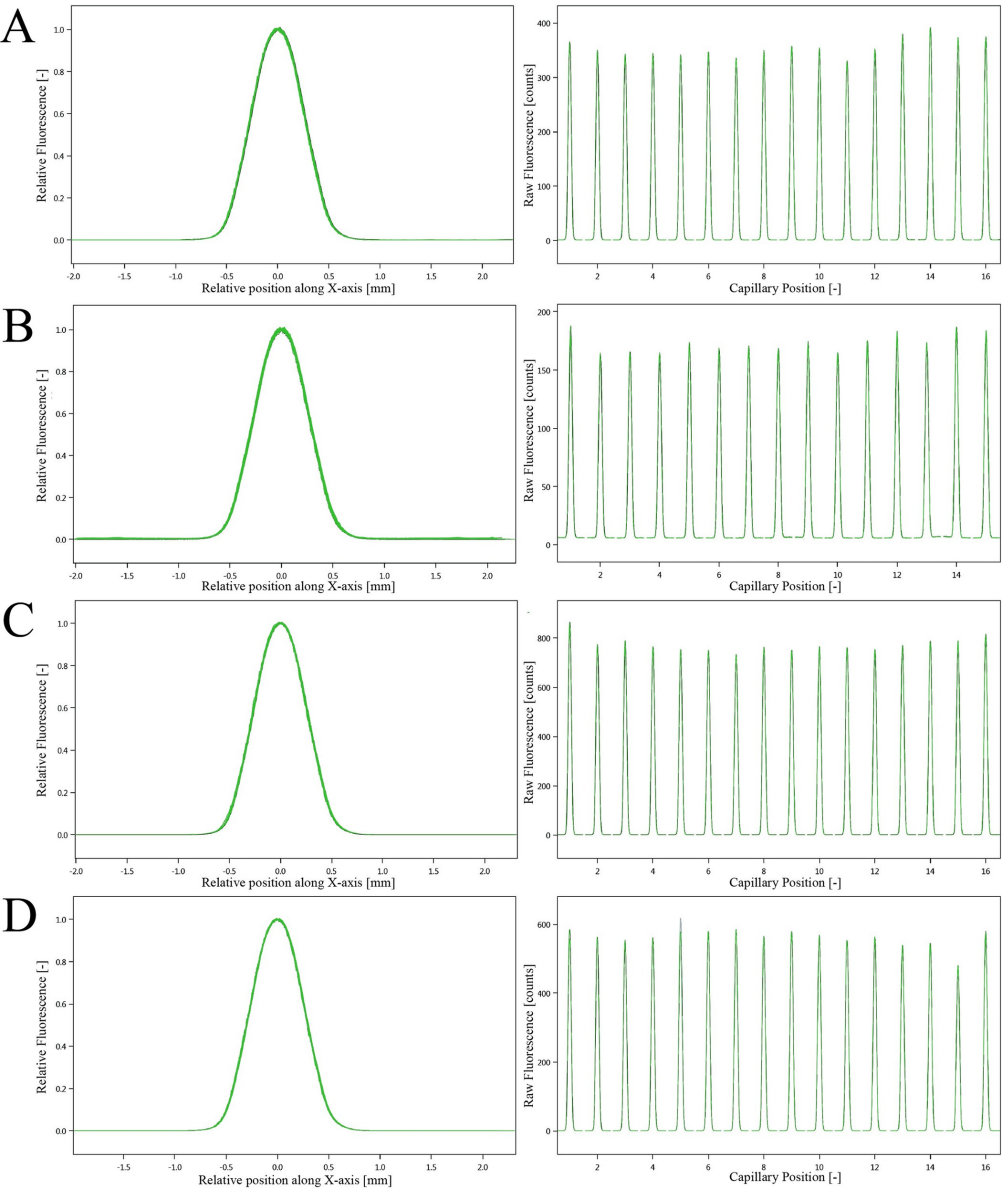
SD-test capillary sharp and SD-test capillary scan (n = 3). (A) Hsp90α (0.945 μM). (B) GPI (0.5 μM). (C) GPD2 (0.02 μM). (D) PGK2 (5.3 μM).

The results of SD-test were shown in Fig 5, and it showed that the effect of protein fluorescent labeling on each protein was negligible, and it would not cause errors in MST detection.

**Fig 6:**
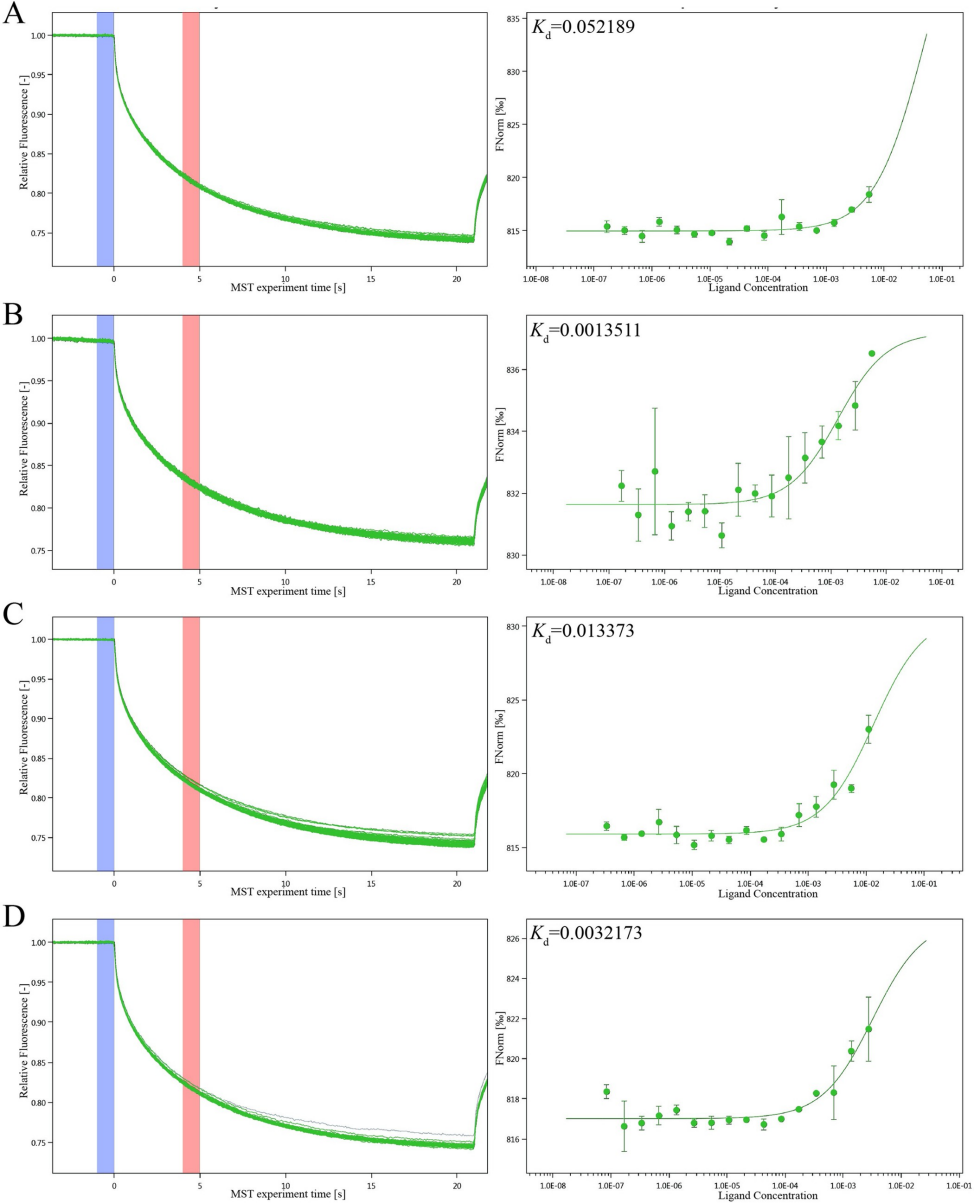
The binding of isoscopoletin with glycolysis-proteins detected by NT.115 analysis (n = 3). MST time traces of isoscopoletin at 16 different concentrations, dependence of the MST signal on the isoscopoletin concentration (measured 30s after turning on heating). (A) Hsp90α (0.945 μM) with isoscopoletin (ranging from 0.000168 to 5.5 mM). (B) GPI (0.5 μM) with isoscopoletin (ranging from 0.000168 to 5.5 mM). (C) GPD2 (0.02 μM) with isoscopoletin (ranging from 0.000336 to 11 mM). (D) PGK2 (5.3 μM) with isoscopoletin (ranging from 0.0000839 to 2.75 mM).

The MST detection was used to detect the affinity of the core proteins selected from molecular docking with different concentrations of isoscopoletin. The dissociation constant *K*_d_ was calculated by the normalized fluorescence difference between bound and unbound states, as shown in Fig 6. It showed that under the condition of given protein concentration of GPD2/0.02 μM, GPI/0.5 μM, Hsp90α/0.945 μM and PGK2/5.3 μM, there was a strong affinity between isoscopoletin and the four core proteins, which suggested that isoscopoletin could strongly bind to GPD2, GPI, Hsp90α and PGK2.

### Effect of antibody on the interaction between isoscopoletin and GPD2

**Fig 7:**
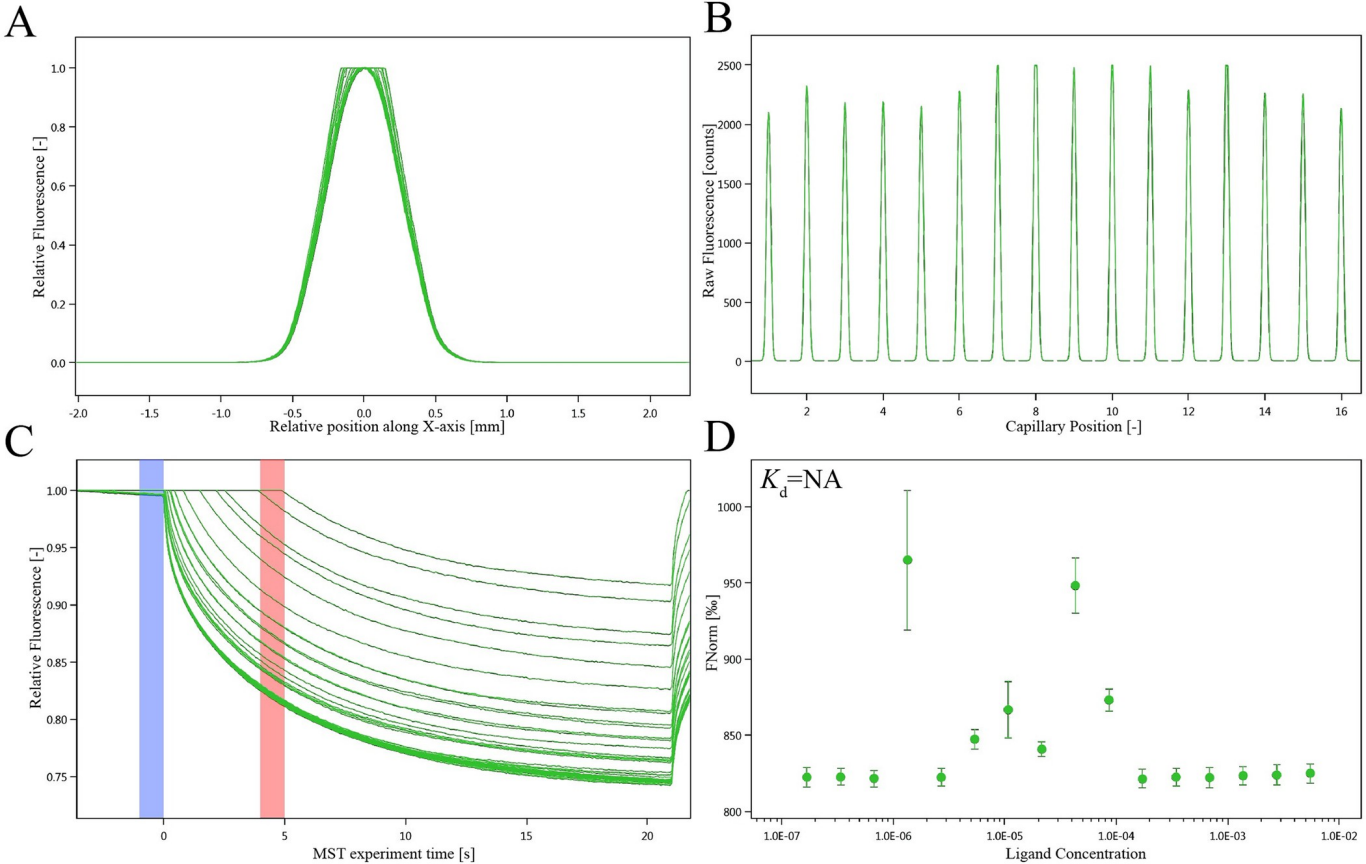
The effect of anti-GPD2 antibody between isoscopoletin and GPD2 (0.5 μM), which was analyzed by NT.115 (n = 3). (A) SD-test capillary sharp. (B) SD-test capillary scan. (C) MST time traces of isoscopoletin at 16 different concentrations (ranging from 0.000168 mM to 5.5 mM). (D) Failed, dependence of the MST signal on the isoscopoletin concentration (measured 30s after turning on heating).

The results of MST detection showed that the antibody could block the binding of isoscopoletin with GPD2, as shown in Fig 7. It indicated that isoscopoletin competed with anti-GPD2 antibody to bind to GPD2.

### Detection of relative cellular viability, glucose consumption and lactate production

**Fig 8:**
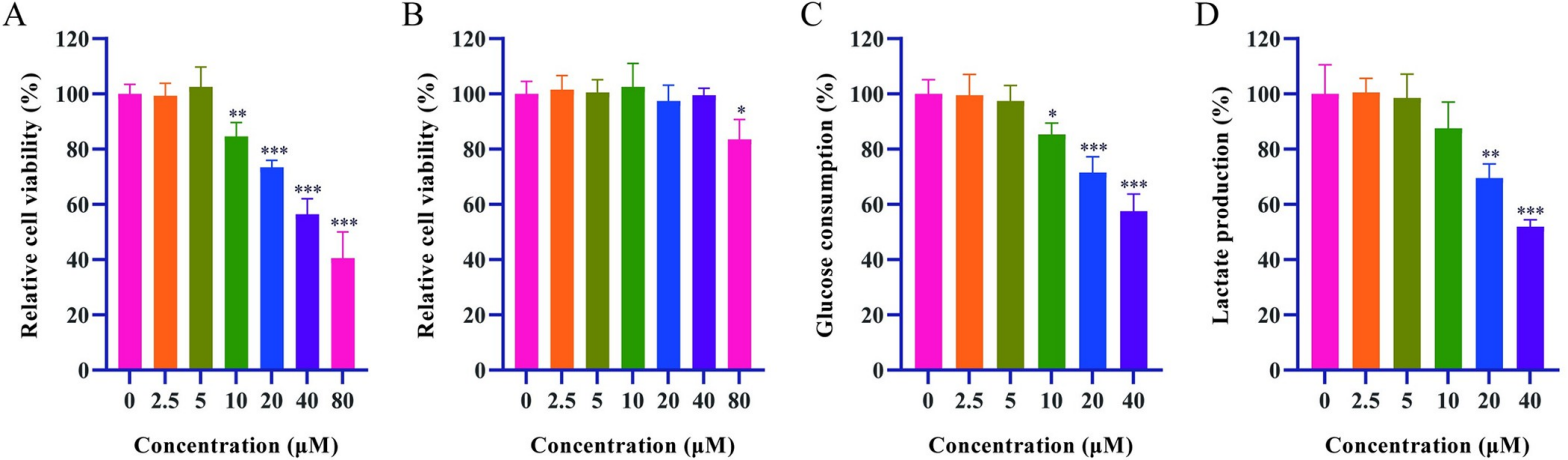
Detection of relative cellular viability, glucose consumption and lactate production after incubation of HepG2 cells with isoscopoletin (n = 3). (A) Effect of isoscopoletin on the activity of HepG2 cells proliferation. (B) The survival of HepG2 cells exposed to 0 to 40 μM isoscopoletin within 1 h. (C-D) Isoscopoletin dose-dependently inhibited glucose consumption and lactate production in HepG2 cells within 1 h. (Data were mean ± SD, *P < 0.05, **P < 0.01, ***P < 0.001).

Fig 8A showed that the proliferation of HepG2 cells began to be significantly inhibited when the concentration of isoscopoletin was 10 μM. The inhibitory effect of enzymes related to the glycolysis pathway could be reflected by changes in intracellular glucose consumption and lactate production. Cells were co-cultured with 0 to 40 μM isoscopoletin for 1 hour to investigate the effect of isoscopoletin on HepG2 cell activity before relative cellular viability, cellular glucose consumption and lactate production were examined. Fig 8B showed that co-incubation of isoscopoletin with HepG2 cells for 1 hour had no significant effect on the activity of HepG2 cells. As shown in Fig 8C and 8D, isoscopoletin inhibited cellular glucose consumption and lactate production in a dose-dependent manner.

### Regulation of isoscopoletin on glycolysis-related mRNA and proteins level

**Fig 9:**
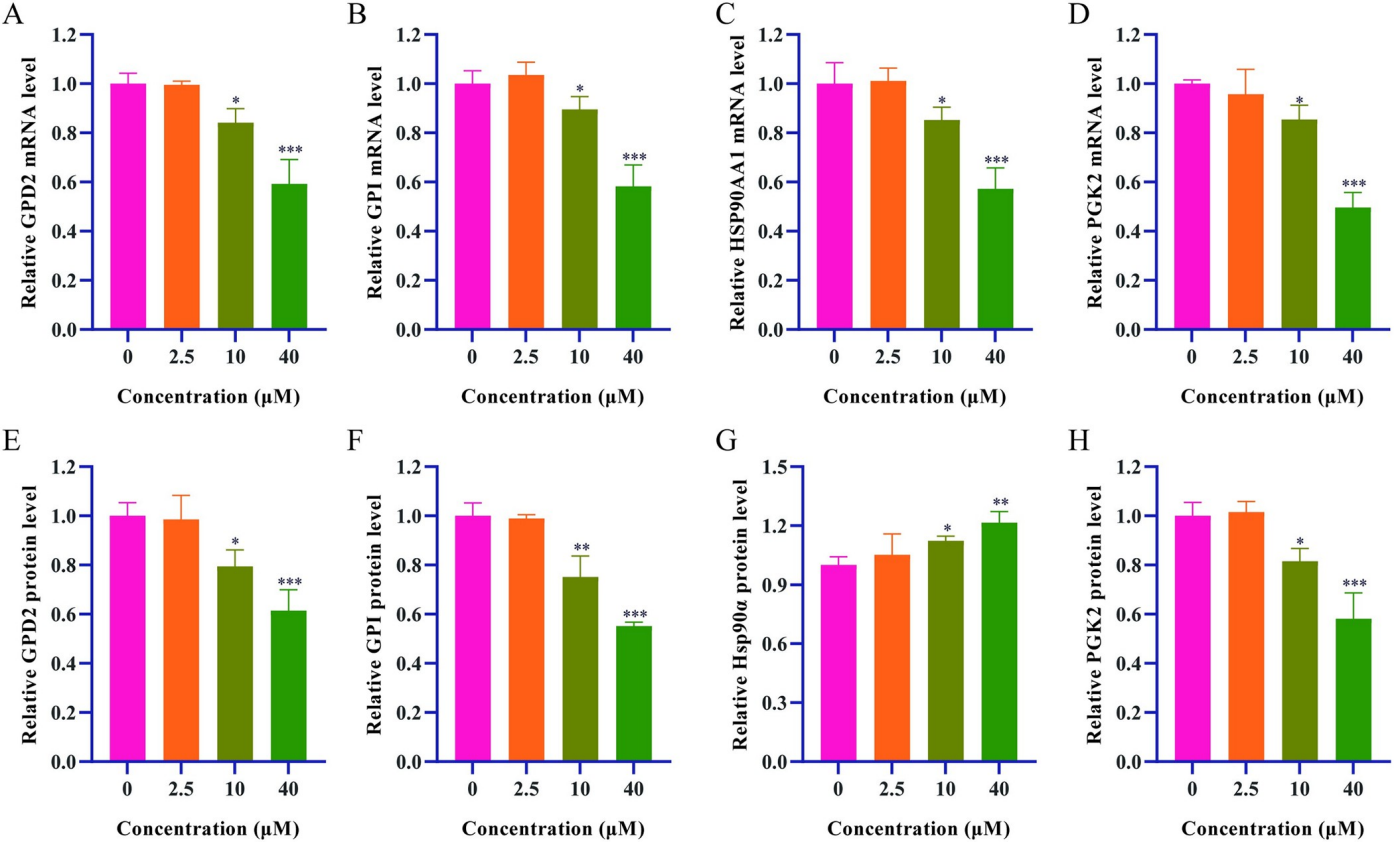
Comparison of mRNA and protein levels in HepG2 cells, 0 μM isoscopoletin group was used as a control (n = 3). (A-D) mRNA levels. (A) GPD2. (B) GPI. (C) Hsp90α. (D) PGK2. (E-H) Protein levels. (E) GPD2. (F) GPI. (G) Hsp90α. (H) PGK2. (Data were mean ± SD, *P < 0.05, **P < 0.01, ***P < 0.001).

The results showed a significant decrease in the mRNA levels of GPD2, GPI, HSP90AA1 and PGK2, along with a significant decrease in the protein expressions of GPD2, GPI and PGK2, as shown in Fig 9. The elevated protein expression of Hsp90α might be due to the reduced rate of glycolysis after its activity was inhibited by isoscopoletin, leading to compensatory expression of Hsp90α in HepG2 cells.

## Discussion

In this study, transcriptome sequencing was used to analyze the DEGs, and the "Isoscopoletin-Target-Pathway" network was constructed through network pharmacology to explore the potential target genes and pathways of isoscopoletin against HCC. Virtual molecular docking and MST were used to verify the binding viability of isoscopoletin to potential targets, and then explore the effect and mechanism of isoscopoletin against HCC *in vitro*. The results of network pharmacology were verified by MST detection and *in vitro* experiments.

After the HepG2 cells were treated with isoscoletin, the DEGs in the extracts were analyzed. KEGG signaling pathway enrichment analysis showed that glycolysis/gluconeogenesis and other metabolic pathways occupied an important position, suggesting that isoscopoletin might play an anti-cancer role through metabolic-related pathways. The results of GO functional enrichment analysis also emphasized that DEGs were mainly involved in the processes related to glucose metabolism.

Based on the results of transcriptomics, the mechanism of isoscopoletin against HCC through glycolysis was explored by network pharmacology and molecular docking. Through the data screening and analysis of network pharmacology, a total of 50 potential target genes and 15 signaling pathways were obtained, which might be the possible information in the anti-HCC process of isoscopoletin. GO functional enrichment analysis found that the potential targets mainly involved in organelle, membrane, synapse and other cell compartments. At the molecular level, the targets were involved in binding, catalytic activity, molecular transducer activity and other molecular activities. The results showed that isoscopoletin might affect the metabolic process and energy utilization by binding to membrane proteins and organelles, thus inhibiting the proliferation of tumor cells. The KEGG signaling pathway enrichment results also indicated that isoscopoletin might play an anti-tumor role by affecting the core targets of glycolysis pathway, such as GPD2, GPI, Hsp90AA1 and PGK2, which were the intersection of most glycolysis-related pathways. Pathways in cancer, central carbon metabolism in cancer, glycolysis/gluconeogenesis, and so on also suggested that isoscopoletin might affect the expression and activity of cancer-related proteins by binding to GPD2, GPI, Hsp90AA1 and PGK2. Through the analysis of network and molecular docking, it was found that isoscopoletin might play an anti-HCC effect by binding to the glycolysis-related proteins. Whether isoscopoletin directly acted on glycolysis-related proteins or not, its binding sites and modes need to be further studied.

Studies have confirmed that lactic acid is generally produced in anoxic conditions, but even when oxygen is abundant, tumor cells will prefer to metabolize glucose to lactic acid to provide energy for themselves. This way of metabolism is considered to be a sign of malignant tumors, a process known as aerobic glycolysis or the Warburg effect [33]. Tumor cells with active metabolism will form a tumor microenvironment rich with nutrient consumption, hypoxia, acidity and toxic metabolites, thus inhibiting the immune system. Glycolysis is accomplished through a series of enzymatic reactions in the cytoplasm. The large number of related enzymes is a direct factor leading to the proliferation of tumor cells in the process of glycolysis. Among them, GPD2, GPI, HSP90AA1 and PGK2 are involved in promoting glycolysis and tumor cell proliferation [5, 6]. These enzymes may be potential biomarkers and potential targets for tumor diagnosis and treatment.

GPD plays a pivotal role in the transfer to aerobic glycolytic tumor cells [34]. GPD1 converts dihydroxyacetone phosphate (DHAP) to Glycerol-3-phosphate (G3P) in the cytoplasm to generate nicotinic adenine dinucleotide (NADH). In contrast, activation of GPD2, which alters its substrate affinity, preferentially converts G3P to DHAP and transforms it into glycolytic catalysis, thus promoting the proliferation of glioma cells [35]. GPI is a key enzyme in glycolysis that catalyzes the reversible reaction of glucose-6-phosphate and fructose-6-phosphate in glycolysis, and this reversible reaction directly affects glycolysis, gluconeogenesis, and gluconeogenesis. oxygen depletion is reversed after GPI knockdown, resulting in reduced glycolytic activity [6]. It has been found [36] that the Warburg effect can be completely inhibited by disrupting the upstream glycolytic enzyme GPI, which forces reprogramming of cancer cells dependent on oxidative phosphorylation thereby controlling breast cancer. In previous studies, GPI was also reported to be upregulated in many cancers, such as lung, gastric and ovarian cancers [37–39]. GPI may play a crucial regulatory role in the development of immune microenvironment and glycolysis in hepatocellular carcinoma cells. Hsp90α is a group of highly conserved and ATP-dependent molecular chaperones that play an important role in cancer cell proliferation, survival and migration by regulating the maturation and stabilization of a variety of oncoproteins [40]. The key functional role as cytoplasmic chaperones is to regulate proteostasis by keeping substrate proteins (such as EGFR, MET, AKT, and many other kinases) in a folded and functional state, thus participating in human cancer development and progression. The overexpression of Hsp90α is 2-10-fold higher in cancer cells compared to normal cells, and cancer cells are highly dependent on Hsp90α chaperone function for proliferation and survival [41, 42]. Yu et al. found that Hsp90α promotes glycolysis thereby accelerating the growth of hepatocellular carcinoma cells [5]. PGK2 is not only a key enzyme in the glycolysis pathway but can be secreted by a variety of tumor cell lines, including pancreatic, breast, and colon tumor cells [43]. The expression of PGK2 in serum was also significantly increased in patients with pancreatic ductal adenocarcinoma compared to the normal group [44]. In previous studies, Esculetin has been shown to exert its inhibitory effect on glycolysis by affecting the activity of PGK2, GPD2 and GPI [27], which provided a valuable reference for the study of isoscopoletin against HCC.

MST detection was used to verify whether isoscopoletin could directly bind to the glycolysis-related targets. MST is a versatile technique to quantify the interactions between molecules, based on the directional movement of molecules in a temperature gradient [45]. The MST results showed that isoscopoletin had a strong directly molecular interaction with GPD2, GPI, Hsp90α and PGK2. And isoscopoletin competed with anti-GPD2 antibody to bind to GPD2, which provided a novel direction for the design of GPD2 binding inhibitors. According to Seidel et al. [24], the fitted curve can be *S*-shaped or its mirror surface. The chemical properties, binding sites and conformational changes after binding led to the standard symbol of MST amplitude. It was suggested that the activation of GPD2, GPI, Hsp90α and PGK2 induced by conformational change might inhibit glycolysis and produce anticancer effect.

MST experiment showed that isoscopoletin could directly bind to glycolysis-related proteins GPD2, GPI, Hsp90α and PGK2. Combined with the results of network pharmacology and molecular docking, it is predicted that isoscopoletin could inhibit the activities of glycolysis-related proteins GPD2, GPI, HSP90α and PGK2, and affect the metabolic process and energy utilization of tumor cells, thus inhibiting the proliferation of tumor cells and exerting its anti-cancer effect.

Further by *in vitro* cellular experiments, it was found that after incubation with isoscopoletin, the relative cellular activity of HepG2 cells decreased, and intracellular glucose consumption and lactate production decreased, along with decreased levels of glycolysis-related genes and proteins, GPD2, GPI, Hsp90α, and PGK2, suggesting that isoscopoletin inhibits the glycolysis process by modulating glycolysis-related proteins, thereby inhibiting HCC cell proliferation, which verified the results of network pharmacology and MST experiments.

## Conclusion

In summary, our results suggest that isoscopoletin plays an anti-cancer role by regulating glycolysis-related proteins GPD2, GPI, Hsp90α and PGK2, and then inhibiting the glycolysis process and proliferation of tumor cells. We believe that our results can provide a better direction and theoretical basis for further understanding of isoscopoletin against HCC.

## Supporting information

S1 Table The information of the 50 intersection targets. (XLSX)

## Abbreviations

BP: Biological process
CC: Cellular component
DEGs: Differentially expressed genes
DMSO: Dimethyl sulfoxide
KEGG: Kyoto Encyclopedia of Genes and Genomes
GO: Gene Ontology
GPD2: Glycerol-3-phosphate dehydrogenase, mitochondrial
GPI: Glucose-6-phosphate isomerase
HCC: Hepatocellular carcinoma
Hsp90α: Heat shock protein HSP 90-alpha
MF: Molecular function
MST: Microscale thermophoresis
PBS: Phosphate buffer saline
PDB: Protein Data Bank
PGK2: Phosphoglycerate kinase 2
PPI: Protein-Protein interaction
